# The efficacy of a scaffold-free Bio 3D conduit developed from human fibroblasts on peripheral nerve regeneration in a rat sciatic nerve model

**DOI:** 10.1371/journal.pone.0171448

**Published:** 2017-02-13

**Authors:** Hirofumi Yurie, Ryosuke Ikeguchi, Tomoki Aoyama, Yukitoshi Kaizawa, Junichi Tajino, Akira Ito, Souichi Ohta, Hiroki Oda, Hisataka Takeuchi, Shizuka Akieda, Manami Tsuji, Koichi Nakayama, Shuichi Matsuda

**Affiliations:** 1 Department of Orthopaedic Surgery, Kyoto University Graduate School of Medicine, Kyoto, Japan; 2 Department of Physical Therapy, Human Health Sciences, Kyoto University Graduate School of Medicine, Kyoto, Japan; 3 Department of Orthopaedic Surgery, Iseikai Yawata Chuo Hospital, Kyoto, Japan; 4 Cyfuse Biomedical K. K., Tokyo, Japan; 5 Department of Regenerative Medicine and Biomedical Engineering Faculty of Medicine, Saga University, Saga, Japan; Szegedi Tudomanyegyetem, HUNGARY

## Abstract

**Background:**

Although autologous nerve grafting is the gold standard treatment of peripheral nerve injuries, several alternative methods have been developed, including nerve conduits that use supportive cells. However, the seeding efficacy and viability of supportive cells injected in nerve grafts remain unclear. Here, we focused on a novel completely biological, tissue-engineered, scaffold-free conduit.

**Methods:**

We developed six scaffold-free conduits from human normal dermal fibroblasts using a Bio 3D Printer. Twelve adult male rats with immune deficiency underwent mid-thigh-level transection of the right sciatic nerve. The resulting 5-mm nerve gap was bridged using 8-mm Bio 3D conduits (Bio 3D group, n = 6) and silicone tube (silicone group, n = 6). Several assessments were conducted to examine nerve regeneration eight weeks post-surgery.

**Results:**

Kinematic analysis revealed that the toe angle to the metatarsal bone at the final segment of the swing phase was significantly higher in the Bio 3D group than the silicone group (-35.78 ± 10.68 versus -62.48 ± 6.15, respectively; *p* < 0.01). Electrophysiological studies revealed significantly higher compound muscle action potential in the Bio 3D group than the silicone group (53.60 ± 26.36% versus 2.93 ± 1.84%; *p* < 0.01). Histological and morphological studies revealed neural cell expression in all regions of the regenerated nerves and the presence of many well-myelinated axons in the Bio 3D group. The wet muscle weight of the tibialis anterior muscle was significantly higher in the Bio 3D group than the silicone group (0.544 ± 0.063 versus 0.396 ± 0.031, respectively; *p* < 0.01).

**Conclusions:**

We confirmed that scaffold-free Bio 3D conduits composed entirely of fibroblast cells promote nerve regeneration in a rat sciatic nerve model.

## Introduction

Treatment of peripheral nerve injury remains challenging, especially in motor nerves with long gaps [1]. Autologous nerve grafting is considered to be the gold standard treatment of nerve injuries that involve an interstump gap [2,3]. However, autologous nerve grafting has several disadvantages, including: limited supply; mismatch of the caliber diameter; and donor site morbidity, such as the loss of native function and neuroma formation [1].

Several alternative treatments for peripheral nerve injury have been developed. For example, some nerve defects have been repaired both experimentally and in clinical practice by bridging the gap with tube-like materials (tubulization) [4,5]. Several nerve conduits using an extracellular matrix [6,7], vascularity [8,9] and supportive cells [10–18] have been developed to improve the quality of regenerated nerves. However, the seeding efficacy and viability of supportive cells injected in nerve grafts remain unclear [19,20]. In addition, the regenerative capacity of nerve conduits remains inferior to that of autografts [21,22]. Furthermore, synthetic nerve conduits are associated with a risk of infection and low biocompatibility [23,24]. To address these potential problems, we focused on the novel technology of Bio 3D printing, and created a completely biological, tissue-engineered, and scaffold-free conduit (Bio 3D conduit) using our novel method to create scaffold-free tubular tissue from homogeneous multicellular spheroids via a Bio-3D-printer-based system. In this system, which utilizes the cellular characteristic of self-assembly, cells cultured in a bioreactor over 24 hours aggregate to form a homogeneous multicellular spheroid structure [25]. Following this aggregation, medical grade stainless needles are used as temporal fixators, called a “needle-array” system, to skewer assembled spheroids until the spheroids are fused. After one week, spheroids are removed and cultured in the bioreactor to obtain a structurally sound scaffold-free construct. This system produces completely biological tubular structures without the need for foreign materials. In the fields of tissue engineering, Bio 3D printing is considered to have several possibilities [25–34].

The purpose of this study was to evaluate peripheral nerve regeneration using the Bio 3D conduit in a rat sciatic nerve model. We used fibroblasts to generate the Bio 3D conduits, because fibroblasts are easy to culture and proliferate in vitro, and it has also been reported that the process of nerve regeneration requires fibroblasts [35]. We confirmed that Bio 3D conduits promote peripheral nerve regeneration based on assessments conducted eight weeks post-surgery in rats receiving Bio 3D conduits compared to the control group using a silicone tube.

## Materials and methods

### Bio 3D conduits

**Fig 1:**
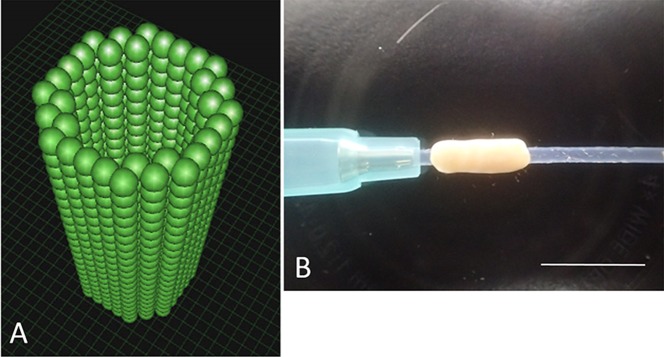
A: The pre-designed 3D tube-like structure. The green spheres represent homogeneous multicellular spheroids that were developed using only human normal dermal fibroblasts. B: The Bio 3D conduit according to the pre-designed 3D model. The conduit was cannulated via a 18-gauge intravenous catheter (SURFLO: NIPRO, Osaka, Japan). Scale bar = 10 mm.

Normal Human Dermal Fibroblasts (NHDF) (Cat No. CC-2509) and monolayer expanded in NHDF medium comprised of Fibroblast Basal Medium (FBM) with Fibroblast Growth supplements (FGM-2) (Cat No. CC-3132) were purchased from Clonetics^TM^ (Lonza, Walkersville, MD, USA). Cells were passaged every 4 days; cells at passage 5 or 6 were used in this study. Conduits were fabricated from NHDF using a Bio-3D Printer (Regenova®, Cyfuse, Tokyo, Japan) as described by Ito et al [27]. Briefly, cells detached and collected by trypsin treatment were centrifuged and re-suspended in a minimal volume of new media. An appropriate amount of cell suspension at a concentration of 3 × 10^5^ cells/mL were incubated in a Low Cell Adhesion 96-well plate (SUMILON PrimeSurface®, Sumitomo Bakelite, Tokyo, Japan). After 24 hours, cells aggregated to form homogeneous multicellular spheroids with diameters of 750 ± 50 μm. To assemble the conduits, spheroids were robotically placed into skewers of a 9 x 9 needle array using the “Kenzan method”; spheroids were arranged in a three-dimensional shape according to a pre-designed 3D model (Fig 1A) by the Bio-3D Printer. Approximately one week after 3D printing, adjacent spheroids were fused to construct a single tubular shape in the Kenzan and the Kenzan was removed. Next, the conduit was transferred to a 18-gauge intravenous catheter (SURFLO: NIPRO, Osaka, Japan) perforated with a 22-gauge needle. The spheroids then were cultured in a perfusion bioreactor to promote self-organization of the living cells until the desired function and strength of the tissue was achieved. Each conduit had a 2-mm internal diameter with wall thickness of 500 μm (Fig 1B).

### Animals

Twelve adult male F344-rnu/rnu rats with immune deficiency (9–10 weeks old, weighing 210–240 g, CLEA, Tokyo, Japan) were used in this study. Rats were randomly divided into the Bio 3D (n = 6) and silicone (n = 6) groups. Each rat was housed in a separate cage, provided food and water ad libitum, and allowed to acclimate to the environment prior to the surgical procedures. This research was approved by the Animal Experimentation Committee, Kyoto University and all experiments were performed in accordance with the Guidelines of the Animal Experimentation Committee, Kyoto University.

### Surgical technique

**Fig 2:**
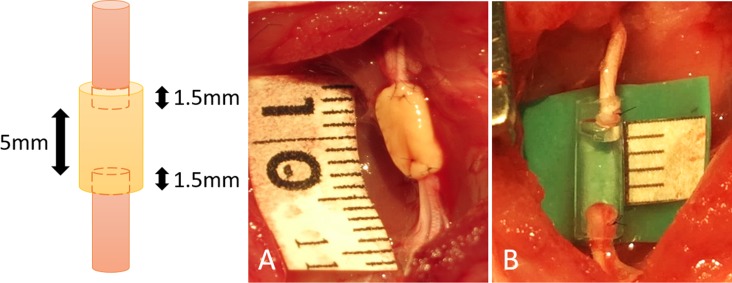
A: In the Bio 3D group, an 8-mm Bio 3D conduit was interposed into the nerve defect, and the proximal and distal nerve stumps were secured 1.5 mm into the tube to create a 5-mm interstump gap in the conduit. B: In the silicone group, the silicone tube with 8 mm length was interposed in the same procedure.

Under general anesthesia (intraperitoneal injection of 40mg/kg pentobarbital sodium with inhalation of isoflurane in oxygen for maintenance), a longitudinal skin incision was made from the right gluteal lesion to the popliteal fossa. The right sciatic nerve of each rat was exposed through a gluteal muscle split and cut in the middle of the thigh to create a nerve defect. An 8-mm Bio 3D conduit was interposed into this region, and the proximal and distal nerve stumps were secured 1.5 mm into the tube to create a 5 mm interstump gap in the conduit (Fig 2A). Both proximal and distal nerve stumps were anchored with 10–0 nylon sutures. The wound was closed in layers with 5–0 nylon sutures. For postoperative analgesia, intraperitoneal fentanyl 0.02 mg/kg was administered. In the silicone group, the silicone tube with 8 mm length and 2 mm internal diameter was interposed in the same procedure (Fig 2B). Following this procedure, animals were allowed to walk freely around their cages and provided with food and water ad libitum. The condition of the animals was monitored every day.

### Pinprick test

The pinprick test was used to evaluate sensory recovery. A pinching stimulus was evoked with standardized forceps by the same examiner for each rat. The right hind limb of the rat was pinched from the toe to the heel until the rat withdrew. The pinprick test was graded from 0 to 3 according to Siemionow et al. as follows: grade 0, no response to stimulus; grade 1, withdrawal response due to stimulus of heel; grade 2, withdrawal response due to stimulus of dorsum of foot; grade 3, withdrawal response due to stimulus of toes [18].

### Toe-spread test

The toe–spread test was used to evaluate motor recovery. The rat is expected to extend and abduct the toes of the uninjured hind limb when suspended by the tail. Toe-spread was graded from 0 to 3 according to Siemionow et al. as follows: grade 0, no toe movement; grade 1, any sign of toe movement; grade 2, toe abduction; grade 3, toe abduction with extension [18].

### Kinematic analysis

To evaluate functional recovery, rats walked on a treadmill to measure the kinematic properties of the hind limbs. Prior to the walking session, rats were equipped with joint markers according to previously described procedures with some modifications [36,37]. Briefly, six landmarks for each hind limb were marked using plastic markers or ink. First, colored hemispheric plastic markers were bilaterally attached onto the shaved skin of five upper landmarks: the anterior superior iliac spine, trochanter major joint (hip), knee joint (knee), lateral malleolus (ankle), and fifth metatarsophalangeal joint (MTP). To detect the toe, acrylic resin ink (paint marker, NIPPONPAINT Co., Ltd, Tokyo, Japan) was applied to the tip of the middle toe. Hind limb motion was captured at a sampling rate of 120 Hz using a 3D motion capture apparatus (Kinema Tracer System, Kissei Comtec, Nagano, Japan) while rats walked at a pace of 10 cm/s. For each rat, a total of 10 steps from sequences in which the rat walked at least 5 consecutive steps was included in subsequent analysis [38]. Markers were traced, and 3D displacements were reconstructed by the system. For the present study, we analyzed 2 parameters: (1) drag toe (DT), the proportion of the step number to the total analyzed step that the rat’s toe was not off the ground; (2) angle of attack (AoA), the toe angle to the metatarsal bone at the final segment of the swing phase. A smaller value on the DT represents less toe dragging. A smaller (sub-zero) value of the AoA indicates that the toe is plantar flexed immediately before the rat’s paw makes contact with the ground.

### Electrophysiological studies

Eight weeks after surgery, the bilateral sciatic nerves were exposed under general anesthesia. The right sciatic nerve was stimulated just distal to the piriformis muscle (S1) and at the popliteal fossa (S2) using an electromyogram measuring system (Neuropack S1 MEB-9404, NIHON KOHDEN, Tokyo, Japan). Two pairs of needle electrodes were inserted into the pedal adductor muscle to check for the presence of compound muscle action potentials (CMAPs) in the muscle. The amplitude (peak to peak) of the CMAPs that were evoked in the pedal adductor muscle with supramaximal electric stimulation from S1 was measured. The distance between S1 and S2 was measured to calculate the motor nerve conduction velocity (MNCV). The same procedure was performed on the left hind limb. The MNCV and the CMAPs in the pedal adductor muscle of the right hind limb were expressed as a percentage of those in the left hind limb.

### Immunohistochemistry

Eight weeks after surgery, CO_2_ euthanasia was performed after the electrophysiological study. The regenerated peripheral nerve was removed from one rat in the each group. After fixation with 4% paraformaldehyde (PFA) and cryoprotection with 20% sucrose, cryostat transverse and longitudinal sections (20 μm thickness) were prepared. After rinsing with phosphate-buffered saline (PBS), antigen retrieval was performed using proteinase K (Sigma-Aldrich, St. Louis, MO, USA) at room temperature for 10 minutes. For blocking, donkey serum was added to the slides, followed by incubation at room temperature for 1 hour. Primary antibody then was added, and sections were incubated at 4°C for 24 hours. Primary antibodies included rabbit polyclonal anti-S100 protein (S-100) antibody (1:1000, Dako Carpinteria, CA, USA) and mouse monoclonal anti-neurofilament H (NF-200) antibody (1:50, Abcam, Tokyo, Japan). Slides then were washed with PBS and incubated with secondary antibody [donkey anti-rabbit IgG (H + L), CF^TM^543 antibody, Sigma-Aldrich; donkey anti-mouse IgG (H + L), CF^TM^488 antibody, Sigma-Aldrich] at room temperature for 1 hour. After further PBS washing, DAPI (4’,6-Diamidino-2-phenylindole, dihydrochloride) solution (1:2000, DOJINDO, Kumamoto, Japan) was added to the slides. After further PBS washing, cover slips were mounted onto the slides using bicarbonate-buffered glycerol (pH 8.6), and slides were viewed using confocal microscopy (BZ-X700; KEYENCE, Osaka, Japan).

### Histological and morphometric studies

Eight weeks after surgery, following electrophysiological study, regenerated nerves were removed from in each group, fixed in 1% glutaraldehyde and 1.44% paraformaldehyde, post-fixed with 1% osmic acid, and embedded in epoxy resin. We prepared transverse sections (1 μm thickness) from the mid-portion of the regenerated nerves. Sections were stained with 0.5% (w/v) toluidine blue solution and examined by light microscopy (Nikon ECLIPSE 80i, Tokyo, Japan). Total myelinated axon number and measurements of the myelinated axon diameter, myelin thickness, and G-ratio were performed using ImageJ software (National Institutes of Health, Bethesda, MD, USA) for morphometric analysis, as reported in our previous studies [8,9,14,39]. Briefly, the total neural area (*a*) of each specimen was calculated by choosing six or seven fields at random so that the area analyzed would represent > 20% of the entire neural area of each specimen. The number of myelinated axons (*b*), neural area (*c*), shortest diameter of each myelinated axon (*d*), and axon diameter (*e*) were calculated for each field at a final magnification of 400×. The number of myelinated axons and neural areas from all analyzed fields then were summed. The total number of myelinated axons in each specimen was estimated as *b* × (*a/c*). The mean myelinated axon diameter was expressed as the average value of the shortest diameter of all myelinated axons in the six or seven fields evaluated. The mean myelin thickness was estimated as (*d-e*)/2 and was expressed as the mean value in the six or seven fields evaluated. The G-ratio was estimated as *e*/*d* and was expressed as the mean value in the six or seven fields evaluated. Ultra-thin sections of the same tissues stained with uranyl acetate and lead citrate were examined using transmission electron microscopy (TEM; Model H-7000; Hitachi High-Technologies, Tokyo, Japan).

### Wet muscle weight of the tibialis anterior muscle

After removing the regenerated nerve, the bilateral tibialis anterior muscles were dissected and detached from the bone at their origin and insertion, and weighed immediately using a digital scale.

### Statistical analyses

Data are presented as the means and standard deviations. Data analyses of the pin prick test and toe spread test were performed with Fisher’s exact test in R (R Foundation for Statistical Computing, Vienna, Austria). Data analyses of the kinematic analysis, electrophysiological studies, the values in morphometric analysis and wet muscle weight measurements were performed with student *t* test in Microsoft Excel 2013 (Microsoft, Redmond, WA, USA). Values of *P < 0*.*05* were considered statistically significant.

## Results

### Pinprick test

No rat was ill or died prior to the experimental endpoint. Eight weeks after surgery, all rats in the Bio 3D group were scored as grade 3 on the pinprick test. In the silicone group, 5 rats were grade 3 and 1 rat was grade 2 (Table 1). There were no significant differences among the two groups.

**Table 1:** The results of the pinprick test and toe-spread test in the Bio 3D and silicone groups.

	Bio 3D group (n = 6)						Silicone group (n = 6)					
No.	1	2	3	4	5	6	1	2	3	4	5	6
Pinprick test	3	3	3	3	3	3	3	3	3	3	2	3
Toe spread test	3	3	3	3	3	3	0	0	2	2	0	0

### Toe-spread test

Eight weeks after surgery, all rats in the Bio 3D group were scored as grade 3 on the toe-spread test. In the silicone group, 4 rats were scored as grade 0 and 2 rats were scored as grade 2 (Table 1). Regarding the toe-spread test, there was significant difference among two groups (*p* < 0.01).

### Kinematic analysis

**Fig 3:**
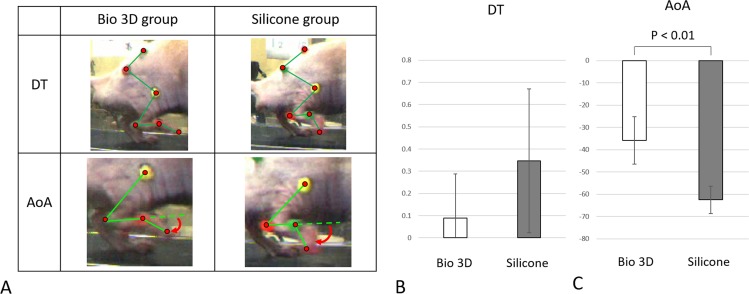
Kinematic studies. A: The photographs demonstrate drag toe (DT) and angle of attack (AoA) in both Bio 3D and silicone groups. In the silicone group, the rat’s toe was not off the ground. The red curved arrows represent the AoA. B: Regarding the DT, there was no significant difference among two groups. C: AoA was significantly different between the two groups (*p* < 0.01). Error bars represent the standard deviation.

The mean DT was 0.089 ± 0.198 in the Bio 3D group, and 0.346 ± 0.324 in the silicone group (Fig 3B). There was no significant difference among two groups. The Bio 3D group exhibited a significantly greater AoA (-35.78 ± 10.68) compared to the silicone group (-62.48 ± 6.15) (*p* < 0.01) (Fig 3C), indicating less plantar flexion of the toe immediately before the rat’s paw made contact with the ground in the Bio 3D group.

### Electrophysiological studies

**Fig 4:**
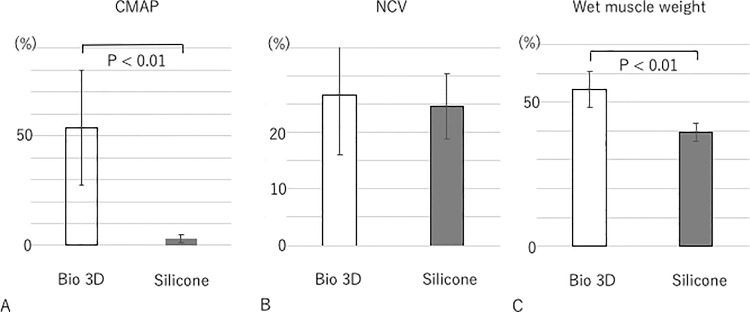
Electrophysiological studies and wet muscle weight of the tibialis anterior muscle eight weeks after surgery. A: CMAP was significantly higher in the Bio 3D group than the silicone group (*p* < 0.01). B: Regarding the NCV, there was no significant difference among two groups. C: Wet muscle weight was significantly higher in the Bio 3D group than in the silicone group (*p* < 0.01). All values are expressed as the percentage of those from the left hind limb. Error bars represent standard deviations.

Eight weeks after surgery, Bio 3D group showed significantly greater mean CMAP compared to the silicone group (53.60 ± 26.36%, 2.93 ± 1.84%, respectively; *p* < 0.01). (Fig 4A). The mean NCV was 26.67 ± 10.6% in the Bio 3D group and 24.69 ± 5.8% in the silicone group. Regarding the NCV, there was no significant difference among two groups (Fig 4B).

### Macroscopic observation

**Fig 5:**
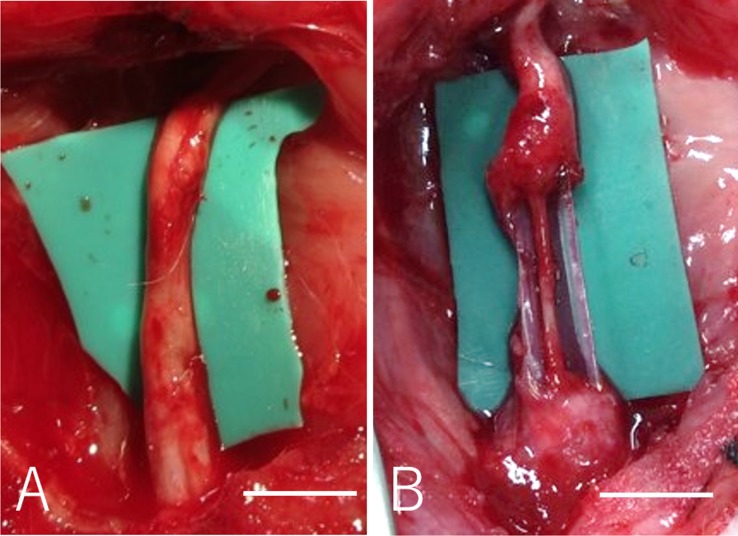
A: Regenerated sciatic nerve eight weeks after surgery in the Bio 3D group. B: In the silicone group, the nerve gap was bridged, however the regenerated nerve was very thin in the silicone tube. Scale bar = 5mm.

In the Bio 3D group, the nerve gap was bridged successfully in all rats, and the degradation of the Bio 3D conduit was confirmed macroscopically eight weeks after surgery (Fig 5A). No neuroma formation was observed. In the silicone group, very thin regenerated nerve was observed in the silicone tube in five rats (Fig 5B) and there was no evidence of neural tissue formation in one rat.

### Immunohistochemistry

**Fig 6:**
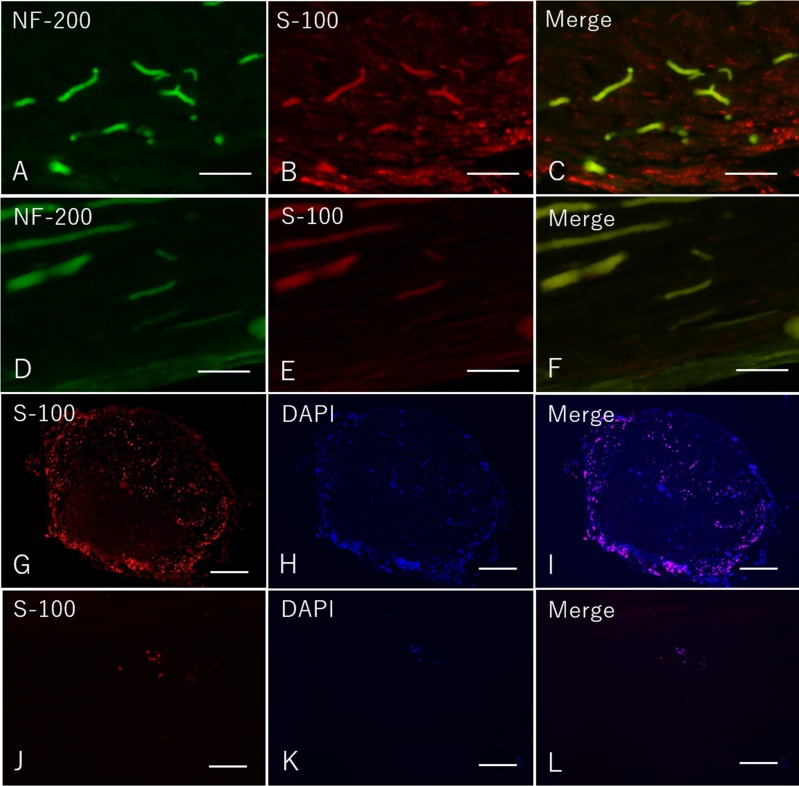
Immunohistochemistry of the mid portion of the regenerated nerve eight weeks after surgery in both groups. A-C: Longitudinal sections in the Bio 3D group. D-F: Longitudinal sections in the silicone group. G-I: Transverse sections in the Bio 3D group. J-L: Transverse sections in the silicone group. A-F: scale bar = 100 μm. G-L: scale bar = 500 μm.

Eight weeks after surgery, immunohistochemical examination revealed that many S-100 and DAPI were expressed in Bio 3D group both in longitudinal and transverse sections (Fig 6A–6C and 6G–6I). In the silicone group, however, slightly expression of S-100 and DAPI was confirmed in both longitudinal and transverse sections (Fig 6D–6F and 6J–6L). These results indicate that the expression of Schwann cells was promoted by the Bio 3D conduit.

### Histological and morphometric studies

**Fig 7:**
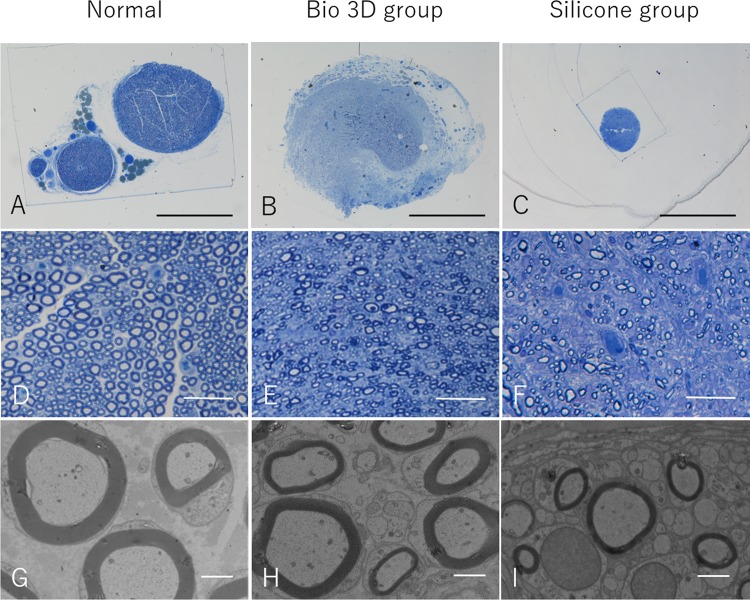
A—F: Semi-thin transverse sections (toluidine blue staining) of the regenerated nerve eight weeks after surgery. A—C: scale bar = 1000 μm. D—F: scale bar = 50 μm. G—I: Transmission electron microscopy of the regenerated nerve eight weeks after surgery. scale bar = 2 μm.

Eight weeks after surgery, semi-thin toluidine blue-stained transverse sections of the mid-portion of the regenerated nerve revealed many well myelinated axons in the Bio 3D group (Fig 7B and 7E). In addition, myelinated axons with proper myelin sheaths were observed in the Bio 3D group under TEM (Fig 7H). The Bio 3D group exhibited a significantly greater myelinated axon number (6516 ± 1694) compared to the control group (2536 ± 1020) (*p* < 0.01) (Table 2). Full morphometric analysis results of the mid-portion of the regenerated nerve in both group and left intact sciatic nerve in the mid-portion of the thigh (normal value) were provided in Table 2.

**Table 2:** Morphometric data eight weeks after surgery in both groups and normal value.

	Normal (n = 5)	Bio 3D group (n = 5)	Silicone group (n = 4)
Myelinated axon number	11351±1900	6516±1694*	2536±1020
Myelinated axon diameter (μm)	4.122±0.291	2.189±0.260	2.146±0.488
Myelin thickeness (μm)	1.112±0.116	0.623±0.072	0.507±0.056
G ratio	0.644±0.013	0.612±0.009	0.649±0.024

Values are expressed as the mean ± standard deviation.

*Bio 3D group exihibited significantly greater myelinated axon number than silicone group (*p* < 0.01).

### Wet muscle weight of the tibialis anterior muscle

Eight weeks after surgery, the wet muscle weight of the tibialis anterior muscle was significantly higher in the Bio 3D group compared to the silicone group (0.544 ± 0.063 versus 0.396 ± 0.031, respectively; *p* < 0.01) (Fig 4C). These results indicate greater muscle atrophy in the silicone group.

## Discussion

We tested the efficacy of a scaffold-free tubular structure developed via a novel Bio 3D printer-based system, the Bio 3D conduit, on peripheral nerve regeneration. To the best of our knowledge, this study is the first to demonstrate the efficacy of a completely biological, tissue-engineered, scaffold-free conduit on peripheral nerve regeneration. This technology may be useful for several neurological disorders, including brachial plexus injuries and severe trauma, in which sources are needed for nerve grafts to treat peripheral nerve defects.

Autologous nerve grafting is considered to be the gold standard treatment for peripheral nerve injuries with an interstump gap [2,3]. Due to several disadvantages of autologous nerve graft, tubulization using nerve conduits has been developed as an alternative treatment for peripheral nerve injury [4,5]. It has been reported that supportive cells, scaffolds, vascularity, and growth factors are essential for peripheral nerve regeneration [40]. Supportive cells (e.g., Schwann cells, bone marrow stromal cells (BMSCs), and fibroblasts) have been utilized to improve the quality of peripheral nerve regeneration [10–18]. Jesurai et al. established a systematic approach to seeding Schwann cells in cold-preserved acellular nerve grafts and reported that the seeding efficacy of supportive cells plays an important role in nerve regeneration [19]. They described that the inner diameters of the needle gauge for injection should be large enough to allow mechanical stress, which leads to the death of the supportive cells; however, a large outer diameter of the needle gauge for injection may cause damage to the epineurium and induce leakage. In addition, to reduce stress on cells, the injection rate can be optimized to deliver the maximum number of viable cells. However, many researchers have reported that the regenerative capacity of nerve conduits remains inferior to that of autografts [21,22]. Furthermore, synthetic nerve conduits are associated with a higher risk of infection and less biocompatibility [23,24]. To improve the seeding efficacy and viability of supportive cells, we utilized novel Bio 3D printing technology to design completely biological scaffold-free Bio 3D conduits. There are two advantages to this novel biological structure: (1) the shape of the conduit has been reported to promote nerve regeneration; and (2) the conduit can deliver viable supportive cells because the conduit material is pure biological material.

Bio 3D printing technology has been used for blood vessels, cartilage, bone, skeletal muscle, bladder, trachea, and myocardium [25–34]. An advantage of this system is that the structure (e.g., size, shape, and length) can be freely designed according to the clinical application using a controlled computer system. Another advantage is that the constructed tissue does not contain foreign materials, which may induce foreign body reactions, infection, or allergy. In addition, the strength of the structure can be controlled by the cell culture period. For example, this technology has been applied to cartilage and trachea, both of which require structural strength [25,28,30]. Indeed, the structure in the present study was strong enough that we could perform the rat sciatic nerve suture using 10–0 nylon.

The process of nerve regeneration requires not only Schwann cells, but also several other cells such as macrophages [41] and fibroblasts [35]. Recent studies have demonstrated that undifferentiated BMSCs (uBMSCs) facilitate nerve regeneration through nerve conduits [10–18]. Implanted uBMSCs produce various types of growth factors and cytokines [13, 42–44], and differentiate into Schwann cell-like cells [14–16, 18, 45–47]. In this study, we used fibroblasts because they are easy to culture and proliferate in vitro, are biodegradable, contain no foreign materials, and exhibit promising mechanical strength. Moreover, it has been reported that the nerve regeneration process requires fibroblasts [35]. Our study confirms that Bio 3D conduits made of fibroblasts only are effective over a 5-mm gap in a rat sciatic nerve model. The methods employed in this study can be applied to construct multilayer Bio 3D conduits composed of multicellular spheroids (MCSs) from uBMSCs or any other cells to improve the seeding efficacy and viability of the cells.

We conducted several assessments to evaluate peripheral nerve regeneration. Motor and sensory nerve recovery was demonstrated in the Bio 3D group via the toe-spread test and pinprick test. In the silicone group, sensory nerve recovery was confirmed via pinprick test, however motor nerve recovery was very poor via toe-spread test. Two rats in the control group exhibited withdrawal in response to a pinprick on the dorsum of the foot (grade 2) because this response reflects an effect of the saphenous nerve, which is the branching of the femoral nerve distributed to dorsum of foot. Electrophysiological studies demonstrated CMAPs in the pedal adductor muscle in the Bio 3D group; furthermore, the mean CMAP was significantly higher in the Bio 3D group than the silicone group. These results indicate that more motor axons are regenerated through Bio 3D conduits compared to the silicone group. Indeed, in the histological study exhibited a significantly greater myelinated axon number compared to the silicone group. We observed the formation of neural tissue in all rats in the Bio 3D group. Immunohistochemistry also revealed NF-200-positive neurofilaments and many surrounding S-100-positive Schwann cells in longitudinal sections. And many S-100-positive Schwann cells and their nuclei also were observed in transverse sections. Histological and morphometric studies revealed many well-myelinated axons in the mid-portion of the regenerated nerve in the Bio 3D group. The wet muscle weight of the tibialis anterior muscle was significantly higher in the Bio 3D group than the silicone group. This result indicates that the Bio 3D group experienced less progression of tibialis anterior muscle atrophy than the silicone group. Kinematic studies revealed a significantly greater AoA in the Bio 3D group. Regarding the DT, there was no significant difference among two groups, however 3 rats in the silicone group revealed over 20% DT. These findings indicate that the sciatic nerve was regenerated to the extent that the tibialis anterior and toe extensor muscles contract against gravity force.

There are several limitations associated with the current study. First, the number of rats in each group was small. Nevertheless, we observed statistically significant improvement in nerve regeneration in the Bio 3D group compared to the silicone group. Second, we did not study control groups receiving autologous nerve grafts. Given that autologous nerve graft is considered the gold standard method of nerve regeneration, future studies should compare the efficacy of Bio 3D conduit to autologous nerve graft. Third, the 5-mm nerve gap is not long enough to assess the efficacy of the Bio 3D conduit. This study did confirm, however, that the Bio 3D conduit promotes nerve regeneration. Future studies should evaluate the efficacy of the Bio 3D conduit for longer gaps (e.g., 10 mm or 15 mm). Fourth, it is not clear how fibroblasts help axonal growth. uBMSCs or Schwann cells should help the axonal growth compare to fibroblasts. Future studies should evaluate the nerve regeneration through the Bio 3D conduit generated by other cells. Finally, the duration of the observation period (eight weeks) after transplantation was insufficient to evaluate nerve function. A longer follow-up period should be considered in future studies.

Additional studies are needed to determine the efficacy of Bio 3D conduits in clinical applications. For example, the mechanisms of degradation of Bio 3D conduits have not been identified. Therefore, a future study should pre-label cells of the spheroids to trace the degraded Bio 3D conduits. A future study also should evaluate the viability of the cells of the spheroids. Furthermore, in the peripheral nerve fields, the mechanical strength and flexibility of the nerve conduit depends on the joint movement. Itoh et al. [27] reported that abundant extracellular matrix was produced in the MCSs, likely originating from fibroblasts, and this extracellular matrix may contribute to the tubular structure. Future studies should evaluate the mechanical strength of the Bio 3D conduits, for example by measuring the bending strength of Bio 3D conduits made from fibroblasts, uBMSCs, and other cells types. Thus, we conclude that the Bio 3D conduit promotes peripheral nerve regeneration and may be useful in peripheral nerve injuries with longer gaps and in clinical applications.

## Conclusion

In the present study, we confirmed that Bio 3D conduits contribute to peripheral nerve regeneration. Further studies of Bio 3D conduits are needed to test their efficacy in clinical applications.
